# Exploring emergency physicians’ knowledge, attitudes, and behaviour towards Choosing Wisely in Taiwan

**DOI:** 10.1371/journal.pone.0271346

**Published:** 2022-07-12

**Authors:** Wang-Chuan Juang, Sonia Ming-Jiu Chiou, Hui-Ling Yang, Ying-Chun Li

**Affiliations:** 1 Department of Quality Management Center, Kaohsiung Veterans General Hospital, Kaohsiung, Taiwan; 2 Department of Emergency Medicine, Kaohsiung Veterans General Hospital, Kaohsiung, Taiwan; 3 Department of Business Management, National Sun Yat‐sen University, Kaohsiung, Taiwan; 4 Institute of Health Care Management, National Sun Yat‐sen University, Kaohsiung, Taiwan; 5 Department of Planning, Kaohsiung Municipal Min-Sheng Hospital, Kaohsiung, Taiwan; ESIC Medical College & PGIMSR, INDIA

## Abstract

**Background:**

In 2012, the American Board of Internal Medicine Foundation launched the Choosing Wisely campaign to reduce unnecessary care. However, it is unclear how much emergency physicians in Taiwan understand about Choosing Wisely. The purpose of this study was to explore the knowledge, attitude, and behaviour of emergency physicians in Taiwan regarding Choosing Wisely and its related factors; the intention was to identify the baseline knowledge on the basis of which to promote Choosing Wisely in Taiwan.

**Methods:**

This was a cross-sectional study including emergency physicians in Taiwan as research subjects who answered online questionnaires. A 42-item questionnaire was designed according to the Knowledge, Attitude, and Behaviour model (KAB). The questionnaire linkages were delivered to emergency physicians through social media (eg., Line, Facebook) and received assistance from different hospital directors. A total of 162 valid questionnaires were collected. Data analyses include t-test, analysis of variance, chi-square test, Pearson’s correlation, and multivariate linear regression model.

**Results:**

The study determined that although only 38.9% of emergency physicians had heard of Choosing Wisely, the mean correct rate of knowledge score among emergency physicians was 70.1%. Attitude and the behaviour related to Choosing Wisely were positively associated, which means that the more positive the attitude towards Choosing Wisely is, the more positive the behaviour towards Choosing Wisely is. In multiple linear regression analyses, having served as a supervisor, belonging to divisions of health insurance service, and having heard of Choosing Wisely (*P* < 0.05) positively affect the knowledge of Choosing Wisely, but age presented a negative association.

**Conclusion:**

This study found that physicians’ knowledge does not influence their attitudes and behaviours, which may be related to barriers of practicing Choosing Wisely activities. To effectively promote Choosing Wisely campaign, it is recommended to focus on the significant factors associated with emergency physicians’ perceptions regarding knowledge, attitude, and behavior of Choosing Wisely. Based on these factors, appropriate practice guidelines for Choosing Wisely can be formulated and promoted.

## Introduction

Many countries have been paying increasing attention to healthcare quality recently. In a setting with limited medical resources, waste produced by unnecessary care affects the quality of healthcare [1]. Unnecessary care may be defined as ‘care provided without a clear medical basis or where the benefits of treatment outweigh the risks’, and it not only increases healthcare costs but also harms patients and the healthcare system [2,3]. Due to time constraints, patient preferences, fear of patient dissatisfaction, patients’ low knowledge of the harms of low-value care, and the unavailability of tools to support shared decision-making, most physicians perform unnecessary care including ordering too many diagnostic tests in the emergency department [4,5].

In 2012, the American Board of Internal Medicine Foundation launched the Choosing Wisely campaign, based on the following four points to promote conversations between clinicians and patients and to eventually reduce unnecessary care: care decisions are to be made (1) on the basis of evidence-based medicine, (2) ordered tests or procedures are to be not duplicative of other tests or procedures already performed, (3) patients are to be kept free from harm, and (4) all procedures are to be truly necessary. Choosing Wisely has been promoted in more than 20 countries worldwide, including Canada, Australia, and Italy [6–8]. Moreover, Hicks et al. found that resource waste considerably decreased after implementing the Choosing Wisely recommendations [9].

Chen et al. indicated that the literature on Choosing Wisely is flourishing globally, but the uptake rate in Taiwan is slow, which indicates that the research on Choosing Wisely is not yet mature [10]. Promoting Choosing Wisely in Taiwan is an opportunity to reduce unnecessary care, save costs, and improve the quality of healthcare [11].

According to Colla et al., understanding the factors that lead physicians to administer unnecessary care is the key to promoting Choose Wisely decision-making [12]. However, emergency physicians are on the frontline of dealing with critically ill patients and are often faced with urgent and complex situations during medical practice. The American College of Emergency Physicians (ACEP) joined the Choosing Wisely campaign in 2013 and published a list of tests and procedures that doctors and patients should question the necessity of, with the goal of reducing administration of low-value tests and procedures [13]. It remains unknown whether emergency physicians understand Choosing Wisely, with relevant research being limited. The purpose of this study was to investigate Taiwanese emergency physicians’ (a) understanding of ACEP’s Choosing Wisely recommendations, (b) their attitudes towards unnecessary care, and (c) their behavioural motivation for administering unnecessary care. The study hypotheses include: 1) respondents’ characteristics have significant associations with knowledge, attitude, and behaviour of Choosing Wisely; 2) knowledge, attitude, and behaviour have significant correlations with each other. We believe our results can help to facilitate the promotion of the Choosing Wisely campaign in Taiwan in the future.

## Materials and methods

This study surveyed emergency physicians in Taiwan. After a literature review and discussion with clinical experts, a 42-item questionnaire was designed according to the Knowledge, Attitude, and Behaviour model (KAB). KAB model is basically based on the theory of planned behaviour states that behavioural modification is manifested in three steps: information acquisition, belief generation and behaviour changes [14–16]. These three steps have been shown to be positively correlated with each other according to previous research [14,16]. The questionnaire is with questions on (1) demographics, (2) knowledge of Choosing Wisely recommendations, (3) attitude towards unnecessary care, and (4) behaviour of delivering unnecessary care. The survey was answered based on a 5-point Likert-type scale. Each construct of KAB has 10 questions. In Knowledge construct, if the respondents provided the right answer, then he/she got one point. The maximum score is 10. In Attitude and Behaviour construct, each of them has 10 questions with 5-point Likert-type scale. Respondents can answer Strong Agree with score of 5, and Strong Disagree with score of 1. The maximum score is 50 for each construct. We invited three emergency physicians to determine expert validity through counting the content validity index, and the pretest questionnaire was distributed from 15 to 28 July 2020. Content validity index (CVI) measures whether a scale is appropriate for the construct of interest. It evaluates whether the domain of content for the construct can be adequately represented by the questionnaire items [17]. We then revised the survey to make the content more readable. A total of 34 pretest questionnaires were received, and the content validity index value was 0.79 which indicates good content validity [17]. We confirmed the applied KAB model’s Cronbach’s α value to be 0.711, indicating an acceptable level of internal consistency. In 2021, there are 2142 emergency physicians specialists approved to give certificates in Taiwan [18]. We set target sample size of 200 which is about 10% of them. We initially planned to apply one month to collect survey but extended the time to get enough responses of survey due to emergency physicians were busy on caring of COVID-19 patients during the study period. The questionnaire linkages were delivered to emergency physicians through social media (eg., Line, Facebook). We also sought assistance from different hospital directors to send questionnaire linkages to emergency physicians. There is no advertisement of the study. Since the responses were all from the online survey, there is no information regarding how many emergency physicians actually clicked the questionnaire linkage in order to estimate response rate. But all of the responses were valid questionnaires. The survey was conducted online between 24 August 2020 and 14 October 2020. We collected a total of 162 valid questionnaires. The study was approved by Kaohsiung Veterans General Hospital’s Institutional Review Board (IRB; IRB number: KSVGH20-CT8-18). The informed consent of the survey respondents was waived through IRB approval. The questionnaire respondents were anonymous.

### Data analysis

We conducted descriptive statistical analysis to determine the distributions of respondents’ characteristics and their perspectives on Choosing Wisely. In the dependent samples, the t-test and analysis of variance were used to analyse the knowledge, attitude, and behaviour scores of Choosing Wisely. The chi-square test was used to examine the correlation between demographics and having heard of Choosing Wisely. Pearson’s correlation was performed to explore the relationship among knowledge, attitude, and behaviour. Furthermore, we applied the multivariate linear regression model to estimate the KAB scores. In our study, all analyses were conducted using SPSS 20.0.

## Results

### Sample characteristics

One hundred sixty-two individuals responded to the questionnaire in this study. Their characteristics were presented in Table 1. Most respondents were emergency specialist physicians (77.2%), and 61.1% of respondents had not heard of Choosing Wisely. Through cross-analysis, we demonstrated that those who had heard of Choosing Wisely were older (*P* = 0.013), had a high education level (*P* = 0.034), were seniors with experience in the emergency department (*P* = 0.005), and had served as a supervisor (*P* = 0.005).

**Table 1:** Characteristics of participating physicians.

Demographic	n(%)
**Gender**	
Male	136 (84.0%)
Female	26 (16.0%)
**Age**	
Under 30 years old	22 (13.6%)
31 to 40	65(40.1%)
41 to 50	49 (30.2%)
51 to 60	25 (15.4%)
Over 61 years old	1 (0.6%)
**Education level**	
graduate school or above	39 (24.1%)
Post-baccalaureate Medicine	5 (3.1%)
Bachelor of Medicine	118 (72.8%)
**Years in practice**	
Less than 10 years	77 (47.5%)
11 to 20	53 (32.7%)
21 to 30	29 (17.9%)
Greater than 31	3 (1.9%)
**Years in practice in emergency dept.**	
Less than 10 years	91 (56.2%)
11 to 20	57 (35.2%)
21 to 30	14 (8.6%)
**Position**	
Chief	27 (16.7%)
VS	104 (64.2%)
R	19 (11.7%)
CR	11 (6.8%)
Fellow	1 (0.6%)
**Have you ever served as a supervisor**	
Yes	106 (65.4%)
No	56 (34.6%)
**Medical institution you used to work(multiple-choice)**	
Medical center	154 (48.7%)
Regional hospital	91 (28.8%)
District hospital	61 (19.3%)
Clinic	10 (3.2%)
**Which level of medical institution is currently working at**	
Medical center	81 (50.0%)
Regional hospital	46 (28.4%)
District hospital	35 (21.6%)
**Divisions of health insurance service**	
Taipei Divisions	33 (20.4%)
Northern Divisions	20 (12.3%)
Central Divisions	32 (19.8%)
Southern Divisions	26 (16.0%)
Kaoping Divisions^a^	48 (29.6%)
Eastern Divisions	3 (1.9%)
**Specialist in Emergency Medicine**	
Yes	120 (77.2%)
No	37 (22.8%)
**Have you previously heard of the Choosing Wisely campaign?**	
Yes	63 (38.9%)
No	99 (61.1%)

VS, Visiting staff; R, Resident; CR, Chief resident.

^a^Kaoping divisions: Kaohsiung and Pingtung county in southern Taiwan.

### Score of knowledge, attitude, and behaviour for Choosing Wisely

On the Choosing Wisely knowledge construct, the mean score was 7.01 (total score of 10), and the mean correct rate was 70.1%. Among topics, ‘avoid CT pulmonary angiography for low-risk patients’ scored the highest.(Table 2). The mean score on the Choosing Wisely attitude construct was 31.82 (total score of 50). Among topics, the attitude that ‘past diagnosis and treatment habits will affect your judgement in implementing unnecessary care’ was the most positive, whereas ‘implement unnecessary care for a patient because of high health insurance reimbursements’ was the most negative. The mean score on the Choosing Wisely behaviour construct was 33.64 (total score of 50). Among topic, the most respondents agreed with ‘I want to rule out the most serious diagnosis, and therefore, I order what would be considered unnecessary tests’, and the fewest respondents agreed with ‘I order unnecessary care because of the high patient insurance reimbursements (including National Health Insurance)’.

**Table 2:** Knowledge of unnecessary care among emergency physicians regarding Choosing Wisely recommendations.

Construct	Questionnaire Item	Mean(SD)
**Knowledge**	1. Head CT (trauma)	0.71 (0.455)
	2.Foley catheter	0.73 (0.446)
	3.Palliative care	0.87 (0.337)
	4.Antibiotics for abscess	0.58 (0.495)
	5.IV fluids	0.76 (0.429)
	6.Head CT (syncope)	0.80 (0.404)
	7.Chest CT (neg D-dimer)	**0.91 (0.291)**
	8.Back pain imaging	0.62 (0.486)
	*9.Antibiotics for abscess	0.51 (0.502)
	10.CT for renal colic	0.53 (0.501)
	Total	7.01 **(**2.00)

CT, computed tomography; IV, intravenous.

*The ninth question is the reverse question.

Table 3 presents the mean scores of respondents on Choosing Wisely’s knowledge, attitude, and behaviour. In the knowledge construct, the mean scores of young respondents, those with less experience in practice, those in the Kaoping divisions of health insurance service, and those who had heard of Choosing Wisely were higher than those of other respondents.

**Table 3:** KAB score for emergency physicians.

	Knowledge (Mean)	P value	Attitude (Mean)	P value	Behaviour (Mean)	P value
**Overall**	7.01		31.8		33.6	
**Gender**		0.09		0.73		0.67
Male	6.89		31.57		28.70	
Female	7.61		31.87		28.34	
**Age**		0.02*		0.04*		<0.001***
<30y	7.36		30.68		27.36	
31-40y	7.39		31.41		27.76	
41-50y	6.89		31.85		28.95	
51-61y	6.03		33.76		31.34	
**Education level**		0.61		0.01**		0.003**
Graduate school or above	7.23		33.48		30.48	
Post-baccalaureate Medicine	6.40		32.80		29.60	
Bachelor of Medicine	6.96		31.23		28.00	
**Years in practice**		0.004**		0.02*		0.001***
Less than 10 years	7.45		31.10		27.71	
11-20y	7.00		31.75		28.56	
21-30y	5.86		33.51		31.00	
Greater than 31y	7.00		35.33		31.33	
**Years in practice in emergency dept.**		0.35		0.03*		0.007**
Less than 10 years	7.19		31.31		28.09	
11-20y	6.84		32.03		28.77	
21-30y	6.50		34.28		31.71	
**Position**		0.75		0.140		0.057
Chief	7.18		33.14		29.81	
VS	6.92		31.71		28.74	
R,CR, and Fellow	7.16		31.06		27.32	
**Ever served as a supervisor**		0.36		0.01*		0.03*
Yes	7.23		31.26		29.66	
No	6.89		32.89		28.11	
**Hospital type**		0.323		0.33		0.86
Medical center	7.20		32.23		28.79	
Regional hospital	6.97		31.73		28.63	
District hospital	6.60		31.00		28.34	
**Divisions of health insurance service**		0.03*		0.055		0.58
Taipei Divisions	7.18		30.63		28.00	
Northern Divisions	6.50		31.30		28.40	
Central Divisions	6.53		30.87		28.21	
Southern Divisions	6.53		32.80		29.73	
Kaoping Divisions	7.75		33.04		28.97	
Eastern Divisions	6.00		30.66		27.33	
**Specialist in Emergency Medicine**		0.61		0.69		0.24
Yes	7.05		31.59		27.97	
No	6.86		31.89		28.84	
**Ever heard of the Choosing Wisely campaign**		0.001***		0.12		0.036*
Yes	7.66		31.43		28.12	
No	6.59		32.44		29.47	

*P < 0.05

**P < 0.01

***P < 0.01.

Total score of knowledge is 10; total score of attitude is 50; total score of behaviour is 50.

### Correlation among knowledge, attitude, and behaviour

Through Pearson’s correlation analysis, only attitude and behaviour in the three dimensions of KAB were determined to have a significant positive correlation (attitude and behaviour: *r* = 0.647, *P* < 0.01). However, knowledge-to-attitude and knowledge-to-behaviour had a less significant correlation.

### Predictors of preference-based scores

We conducted multiple regression analysis on three dimensions to evaluate the predictive indicators of emergency physicians’ scores on Choosing Wisely’s knowledge, attitudes, and behaviours. The results indicated that age, having served as a supervisor, working in the health insurance division, and having heard of Choosing Wisely were predictors of knowledge score. On the other hand, age had a negative influence, meaning that older age was correlated with lower score for Choosing Wisely knowledge. The *R*^2^ of this model was 0.261 (Table 4). However, the results of the multiple regression models of attitudes and behaviours were not significant.

**Table 4:** Predictors of emergency physicians’ scores on KAB of Choosing Wisely.

Dependent variable:	Knowledge score			Attitude score			Behaviour score		
	Coef	SE	P value	Coef	SE	P value	Coef	SE	P value
Independent variable									
**Gender (ref: Female)**									
Male	-0.43	0.407	0.28	-0.015	0.090	0.86	0.000	0.061	0.99
**Age**	-0.14	0.034	<0.001**	0.007	0.008	0.33	0.008	0.005	0.10
**Education level (ref: Bachelor of Medicine)**									
Graduate school or above	0.28	0.396	0.47	0.12	0.087	0.14	0.14	0.059	0.01**
**Years in practice in emergency dept.**	0.028	0.035	0.43	0.001	0.008	0.84	-0.002	0.005	0.76
**Position(ref: Chief)**									
VS	0.45	0.515	0.37	-0.02	0.113	0.86	-0.013	0.077	0.86
R,CR, and Fellow	-0.35	0.753	0.64	0.041	0.166	0.80	0.063	0.113	0.57
**Ever served as a supervisor(ref: No)**									
Yes	1.23	0.461	0.008**	0.031	0.102	0.76	-0.075	0.069	0.27
**Hospital type (ref: district hospital)**									
Medical center	0.022	0.428	0.95	0.15	0.094	0.11	0.039	0.064	0.54
Regional hospital	0.037	0.433	0.93	0.08	0.095	0.36	0.015	0.065	0.81
**Divisions of health insurance service(ref: Taipei Divisions)**									
Northern Divisions	-0.18	0.542	0.73	0.053	0.119	0.65	0.045	0.081	0.57
Central Divisions	-0.21	0.496	0.66	0.031	0.109	0.77	-0.002	0.074	0.97
Southern Divisions	0.034	0.514	0.94	0.19	0.113	0.09	0.037	0.077	0.63
Kaoping Divisions	0.87	0.437	0.048*	0.17	0.096	0.06	0.048	0.065	0.46
**Specialist in Emergency Medicine(ref:No)**									
Yes	0.10	0.469	0.81	0.043	0.103	0.67	0.005	0.070	0.94
**Ever heard of the Choosing Wisely campaign(ref: No)**									
Yes	1.07	0.316	0.001**	0.005	0.070	0.94	-0.016	0.047	0.73
**R** ^**2**^		0.26				0.14			0.09

*P < 0.05

**P < 0.01.

## Discussion

### Statement of principal findings

This study aimed to investigate the knowledge, attitudes, and behaviours of emergency physicians in Taiwan regarding Choosing Wisely. The results showed that only 38.9% of the emergency physicians in Taiwan surveyed had heard of Choosing Wisely, indicating that Choosing Wisely campaign was not well-known in Taiwan. Our results found that attitude and behaviours are positively correlated; however, knowledge does not influence attitudes and behaviours.

### Strengths and limitations

In this study, we investigated the perspective of emergency physicians about Choosing Wisely in Taiwan, which was limited reported in previous studies. This study highlights the important factors affecting emergency physicians to implement Choosing Wisely campaign. It will facilitate a better reduction of ordering unnecessary care. However, this study has three limitations. First, this study used questionnaires, and the study results cannot represent the true clinical behaviour of the physicians. In addition, the interviewees voluntarily participated, which means their statements cannot represent the views of all emergency physicians in Taiwan. Additionally, the questionnaire is filled out according to personal interests and the results of the research may be overestimated. Second, only 162 samples were collected in this study, which may have been insufficient. In 2021, there are 2142 emergency physicians specialists approved to give certificates in Taiwan [18]. To verify our sample size is enough for the study, we estimated the sample size by applying a confidence level of 95%, assuming a 5% margin of error, a population frequency of 10%, and population size of 2142. A minimum required sample size of 130 participants was calculated [19]. Therefore, our study sample size is valid for the study. Most of the samples were collected from physicians working in medical centres and working in the southern region. Therefore, the results were possibly affected by respondents being from different levels of hospitals and health insurance districts. Because the survey samples were not through randomly selection, it cannot claim that the study participants are representative of other emergency physicians in Taiwan. Therefore, it must be cautioned to consider external generalizability of the study results. Finally, this study only examined emergency physicians’ knowledge, attitude, and behaviour regarding Choosing Wisely and did not investigate the difficulties of emergency physicians in implementing Choosing Wisely guidelines in clinical practice.

### Interpretation within the context of the wider literature

The mean correct rate of physicians in the knowledge construct of Choosing Wisely was 70.1%, which was consistent with the results in the United States and Spain [20,21], indicating that emergency physicians in Taiwan have sufficient knowledge of evidence-based medicine. However, Choosing Wisely is still not prevalently promoted in Taiwan, which indicates that there is a need to strengthen the understanding of the benefits of implementing Choosing Wisely.

Our results suggest that ‘past diagnosis and treatment habits’ may be a major factor in the attitude of emergency physicians towards unnecessary care. The rule of thumb for physicians is to make decisions based on previous clinical experience; this is a cognitive bias. Studies in Brazil have yielded similar results to ours [22,23]. Levinson’s research demonstrated that most people are reluctant to change old habits, and thus, a new approach needs to offer simplified or relatively beneficial results for it to be adopted [8]. Some have recommended using targeted interventions such as audit and feedback and clinical decision support to alert physicians when their decisions deviate from clinical guidelines [24–26] or presenting to physicians the positive effects of implementing Choosing Wisely, which may help improve physicians’ attitudes toward Choosing Wisely. Attitude is expected to affect behaviour. Attitude contains an individual’s opinion (belief/disbelief) or emotional response (liking/disliking) towards an object. Behaviour consists of actions or observable responses that are the result of an attitude [27].

We also found that physicians were influenced by the behaviour of ‘want to rule out (R/O) serious diagnosis’ to administer unnecessary care. This result is consistent with those of a survey of emergency physicians in the United States. Because decision-making rules are imperfect or not completely understood, physicians may be slow to adopt clinical guidelines in clinical practice [28]. Moreover, being concerned about failing to make a diagnosis is considered an aspect of medical culture that is difficult to change [29]. Therefore, the participation of medical providers, management support, cultural reforms, clear data and training, and teamwork may help narrow the gap between guidelines and practice, leading to more beneficial outcomes [30].

In terms of attitude and behaviour, emergency physicians in Taiwan did not agree that ‘insurance benefits’ would affect the implementation of unnecessary care, indicating that emergency physicians in Taiwan are not tempted by the advantage of insurance benefits. This result has also been verified in Charlesworth’s (2014) and Colla’s (2018) studies. Low-value medical care is not related to insurance types or expected insurance benefits [31,32].

The significant effect of age on the knowledge score of Choosing Wisely indicates that younger physicians are more knowledgeable about evidence-based medicine than older physicians. Similar results have been found in past studies, which can be explained by the more up-to-date academic training of younger physicians [21]. For example, Taiwan developed ‘post-graduate year training, PGY’ in 2003, adding evidence-based medicine to medical education; therefore, younger physicians have greater knowledge [33]. By contrast, on the attitude and behaviour score of Choosing Wisely, older age, higher education level, longer practicing experience, longer working experience in the emergency department, and serving as a supervisor were higher than other respondents. We can infer that senior physicians in the emergency department were more aware of the need to reduce unnecessary care. Although younger physicians exhibited greater knowledge, senior physicians performed better in attitude and behaviour, indicating that knowledge is not reflected in attitude and behaviour. The reason may be that younger physicians lack time to follow new decision making methods such as Choosing Wisely due to heavy workload, or they order unnecessary diagnostic tests because of the fear of making wrong decisions and having little experience.

### Implications for policy, practice and research

Exploring emergency physicians’ understanding of Choosing Wisely can help evaluate development strategies. Taiwan’s Choosing Wisely is still in the promotion stage. Much unnecessary care is delivered in Taiwan. For example, according to previous studies, the use of antibiotics is very common in Taiwan, and inappropriate prescription of antibiotics can increase the risk of potential adverse effects, incur unnecessary costs, and lead to antibiotic resistance [34,35]. Another example, the elderly with insomnia, anxiety, depression, and other mental disorder were more likely to receive benzodiazepine prescriptions. However, inappropriate use of benzodiazepine may be associated with the risk of fractures [36,37]. It is an opportunity to reduce unnecessary care, save costs, and improve the quality of healthcare through promoting Choosing Wisely in Taiwan [11]. Healthcare policy maker can formulate clearer Choosing Wisely practice guidelines. From 2016, a series of forum discussions of evaluating low-value cares initialized Choosing Wisely Taiwan campaign. Through systematic literature review and discussing with experts, Cochrane Taiwan then suggested five top low-value cares that need to be improved as top priority. In 2020, Joint Commission of Taiwan recommended hospitals to take actions to avoid low-value care and reduce unnecessary resource wastes, and suggested hospitals can follow US Choosing Wisely as references and added it into hospital accreditation item [38,39]. This will lead hospitals to incorporate Choosing Wisely into hospital policies to change physicians’ habits of performing medical treatment and use relevant education and training and related tools, such as the shared decision-making and clinical decision-making system of medical and disease, etc., to improve medical waste and improve the quality of medical treatment.

## Conclusion

This study demonstrated that the prevalence of Choosing Wisely adherence is still low in Taiwan. Emergency physicians have sufficient knowledge of practical medicine, but entrenched diagnostic habits and the desire to rule out serious diagnoses continue to lead to delivery of unnecessary care. More effort needs to be made to promote Choosing Wisely in conjunction with the adoption of relevant interventional policies and teamwork.
